# Anti-Vivisection Methods

**Published:** 1902-07-12

**Authors:** 


### ANTI-VIVISECTION METHODS.

We have received from the Hon. Stephen Coleridge the following letter:—

The National Anti-Vivisection Society,
92 Victoria Street, London, S.W.,
July 4, 1902.

Sir,—The conclusion of your article entitled "Anti-Vivisection Methods," on p. 245 of The Hospital for July 5th, conveys, no doubt unintentionally, a totally false impression of the action of the jury in the recent case of Coleridge *v.* Macdonald.

It is true that in my opinion as expressed in the witness-box, Mr. Macdonald's letters amounted to accusing me of being a liar, and were therefore damaging to my character, and you are good enough to say that you share my opinion, but you are quite wrong in suggesting that the jury took the same view: if they had, the letters would have been libels and I should have won my case. As a fact they came to the conclusion, not that the letters implied that I was a liar and were justified on the facts, but that the letters did not imply that meaning, and were, therefore, not libellous. The defence were satisfied to get a verdict on these grounds and did not plead justification nor make any attempt to prove that I was a liar.

I am glad to find that you disagree with both the judge and the jury, and take the same view of the letters that I did.

My chief object in bringing the action was to afford my adversaries the opportunity of cross-examining me on my anti-vivisection methods, and of proving, if they could, that I ever transgressed the lines of truth and honour in my controversies, but they did not venture into that arena by accepting my and your interpretation of the letters and by pleading justification.

I appeal with confidence to your sense of fairness to insert this letter in your next issue and so correct the very inaccurate impression that is placed before your readers in your article.

Your obedient servant,

Stephen Coleridge.

Whatever may have been Mr. Coleridge's object in commencing the action for libel to which he refers, he can hardly hold, we should imagine, that the defendant was called upon to afford facilities for a discussion as to the veracity of the statements made from time to time in the name of the Anti-Vivisection Society. Indeed, we believe that if Mr. Coleridge's counsel had let out at the beginning of the trial that it was with this object that the action had been brought, the Judge would pretty quickly have made it clear that he would not allow his Court to be made a cock-pit for any such disputation. But if one refers to the opening statement of the counsel for the plaintiff, one finds that Mr. Coleridge was by no means so anxious to come into Court as he implies, for it is there stated that not only did the plaintiff's solicitors make "an offer to the effect that no action would be brought on the defendants publishing an apology and paying costs up to date" (we quote from the account of the trial published in the *British Medical Journal*, June 21st, 1902), but that, later on, they wrote to the defendant's solicitors stating that if the defendant was prepared to publish an apology the plaintiff would be prepared to withdraw his action, and ask nothing for costs, from which we may opine that Mr. Coleridge had not disclosed his ardent desire to be cross-examined even to his own solicitors.

Passing on, however, to the point which Mr. Coleridge more especially raises in regard to ourselves, we must state at once that while he is perfectly correct in stating that the verdict, which went against him, was to the effect that the words written were not libellous, his suggestions in regard to the line of argument which led to this verdict are mere speculations, and that he has no right to jump to the conclusion that because the jury stopped the case they meant that the defendant had not, as Mr. Coleridge puts it, "accused him of being a liar." Mr. Coleridge's own counsel quoted the words of Lord Bowen to the effect that "a man has a right . . . to express his opinion provided that he does not go beyond the limits which the law calls 'fair,' and although we cannot find in any case an exact and rigid definition of the word 'fair,' this is because the judges have always preferred to leave the question of what is fair to the jury." Thus the question for the jury to decide was not, as Mr. Coleridge seems to imagine it was, whether or not Mr. Macdonald had accused him of being a liar, but whether what Mr. Macdonald said was "fair," and when the jury by their verdict said there had been no libel, they did not express any opinion upon the veracity of Mr. Coleridge, nor give him any testimonial on the subject.

We believe we are right in saying that in the determination of a question of libel, the character of the person libelled and the circumstances of the controversy, as well as the words uttered, have to be taken into consideration, and in regard to this we must quote the remarks made by Mr. Justice Grantham, who is reported to have said, "The jury have expressed an opinion, and I agree with them. Mr. Coleridge sends letters to me and to other members of the legal profession every month on the subject of vivisection. He hits hard in controversy, and people who hit hard must not be too thin-skinned. The jury wish to add a rider to the effect that in their opinion this action never ought to have been brought."

Now if Mr. Coleridge is at liberty to exercise his imagination in regard to what led the jury to give their verdict, others may do the same, and it is evidently open to them to believe that the jury regarded the action under the circumstances as frivolous. But to what depths must controversy have descended, and what is to be thought of anti-vivisection methods when a man accused, as Mr. Coleridge says he was accused, is told from the bench that he "must not be too thin-skinned!"

